# The mediating effects of attitude toward death and meaning of life on the relationship between perception of death and coping with death competence among Chinese nurses: a cross-sectional study

**DOI:** 10.1186/s12912-023-01245-5

**Published:** 2023-03-30

**Authors:** Shijia He, Hong Zhao, Huiping Wang, Fengzhi Chen, Tingting Lv, Lezhi Li, Huilin Zhang

**Affiliations:** 1 Clinical Nursing Teaching and Research Section, Second Xiangya Hospital, Central South University, Changsha, Hunan China; 2 Xiangya School of Nursing School, Central South University, Changsha, Hunan China; 3 School of Nursing, Hunan University of Chinese Medicine, Changsha, Hunan China

**Keywords:** Perception of death, Competence to cope with death, Attitude toward death, Meaning of life, Mediating effects, Nursing, Death education

## Abstract

**Background:**

It is important to understand how the perception of death affects the competence to cope with death.

**Objectives:**

To explore whether the perception of death has an indirect effect on competence to cope with death through the mediation of attitude toward death and meaning of life.

**Methods:**

A total of 786 nurses from Hunan Province, China, selected by random sampling method and asked to complete an online electronic questionnaire between October and November 2021 were included in the study.

**Results:**

The nurses’ scored 125.39 ± 23.88 on the competence to cope with death. There was a positive correlation among perception of death, competence to cope with death, the meaning of life, and attitude toward death. There were three mediating pathways: the separate mediating effect of natural acceptance and meaning of life, and the chain mediating effect of natural acceptance and meaning of life.

**Conclusion:**

The nurses’ competence to cope with death was moderate. Perception of death could indirectly and positively predict nurses’ competence to cope with death by enhancing natural acceptance or sense of meaning in life. In addition, perception of death could improve natural acceptance and then enhance the sense of meaning in life to positively predict nurses’ competence to cope with death.

## Introduction

With the development of aging population, the change of disease spectrum and the prominence of excessive medical problems, hospice care has gradually attracted attention and become one of the major global health issues. Hospice care originated in the UK, which has always been in the leading position in the world, with a high level of national awareness and participation, and a well-developed system [1]. The hospice care in European and American countries started early and has become an important part of the social medical and health care system [2]. Hospice care has made rapid progress in some East Asian countries, such as Japan and Korea, although it started late [3, 4]. China has promoted the development of hospice care through economic and policy approaches, but it has not yet formed a good development trend and still cannot fill the huge demand gap [5]. A 2015 Economist Intelligence Unit (EIU) report showed that mainland China ranked only 71 out of 80 countries and territories in terms of quality of death [6]. Death education is one of the basic and important parts of hospice care, yet it is still not widely available in China. The importance of nurses in death education and hospice as implementers of hospice care, promoters of death education, and one of the populations most exposed to death cannot be overstated. Nurses’ coping with death competence (CDC) includes their competence to deal with death-related events, such as communicating with bereaved families, effectively dealing with negative emotions caused by death events, and providing hospice care for patients [7]. A study found that 33.33% of the nurses did not cope well when they faced the death of the patient [8]. Findings in China also showed that nurses were less well prepared in coping with death [9]. Poor CDC has been showed to increase nurses’ burnout, compassion fatigue, negative emotional distress and job dissatisfaction, which ultimately affects the quality of care for patients [10, 11]. Against the backdrop of the current aging population and COVID-19 epidemic, a high level of CDC among nurses is particularly required [12]. Therefore, it is essential for nursing managers and educators to investigate the risk factors and influencing mechanisms associated with nurses’ CDC, which may provide a basis for developing effective and targeted strategies.

## Background

### Perception of death and CDC

As clinical staff who contacts most frequently with end-stage patients and their families, and who is most exposed to death, nurses play an important role in hospice care [13]. CDC is one of the important professional competencies of nurses and the key to the quality of hospice care; it is closely related to burnout, compassion fatigue, job satisfaction, and personal quality of life [14–16]. It can be said that nurses’ CDC is of great value to clinical care, and to individual nurses as well as patients and their families. Studies have shown that a considerable number of nurses experience difficulties in communicating with the families of dying patients and lack end-of-life communication skills, which is one of the manifestations of the lack of CDC [17]. Besides that, nurses go through a range of emotions after the death of a patient such as fear, guilt and self-blame [18]. Their CDC can be affected by various factors such as age, work experience, attitude toward death, self-care ability, death education experience and social support [14, 19, 20].

The study showed that nurses who had received death education had higher overall CDC than those who had not. Possibly because these courses provided knowledge of coping with dying and death, as well as opportunities to discuss death-related events, which improved nurses’ knowledge and skills in coping with death, and finally their CDC [21]. In Chinese tradition, the cultural convention of “How can you know about death before you figure out the purpose of living?“ makes “death” seldom a topic of conversation among Chinese people. Most of them treat “death” as an unlucky word, making the development of death education in China very challenging. Although with the development of hospice care in the world, death education in China wins increasing attention in recent years, it still develops relatively slow due to its late start, imperfect curriculum system, lack of corresponding teaching materials and teachers [13]. Current death education for nurses is mostly in the form of short lectures focusing on cognitive aspects such as pain control, symptom management, and communication skills. It is inadequate to meet the high-level demand of death education in nurses’ CDC [22, 23], hospice care and practical clinical work. To solve this problem and improve nurses’ ability to handle death events, scholars advocate the development of individualized and diverse death education. However, current research still lacks the exploration of the mechanism of death perception and CDC, as well as the theoretical development of reasonable and effective death education strategies.

### The potential mediating effect of attitude toward death

Attitude toward death refers to the individual’s stable and evaluative psychological tendency towards death, including negative attitudes such as death anxiety, death fear, and death avoidance, as well as positive attitudes such as natural acceptance, approach acceptance, and escape acceptance [24]. Researches have shown that nurses accept the phenomenon of death as a natural process of human life; however, they continue to suffer from death fear and death avoidance, which was particularly more obvious during the COVID-19 pandemic [25, 26]. According to the results of a study, the vast majority of respondents agreed with the statements that death is an unpleasant experience (63%) and nurses’ death fear score is a whopping 5.3 (maximum score of 7) [26]. An individual’s attitude toward death often depends on his understanding of death [27]; therefore, the death education for nurses can influence their attitudes toward death and caring for dying patients [2]. The theoretical model of Knowledge, Attitude/Belief, Practice (KAP) [28] divide the change in human behavior into three continuous processes: acquiring knowledge (Knowledge), generating belief (Attitude), and forming behavior (Practice). Behavioral change is based on Knowledge, while its driving forces are belief and attitude. Nurses’ behavior of caring for dying patients may be affected and changed by their attitudes towards death [29]. A previous study revealed that the more the nurses understood the concepts related to death, such as hospice care, death, and euthanasia, the more positive their attitude toward death was [30]. Positive death attitudes such as natural acceptance, approach acceptance, and escape acceptance are positively associated with nurses’ CDC [31]. Nurses with a negative attitude toward death had difficulties in providing spiritual care and mental care [32]. Studies have shown that nurses with high levels of death avoidance and death fear are significantly less competent in dealing with dying or dead patients and their families, meaning that negative death attitudes may be detrimental to the CDC [31, 33]. Therefore, attitude toward death may play a mediating role between death perception and CDC.

### The potential mediating effect of meaning of life

Death is a part of life. It is with the existence of death that life has a time limit, which is particularly valuable. People think about death; they realize that every life is living toward death, and then further reflect on the existence of human beings and the meaning of life [34]. The sense of meaning in life refers to people’s subjective evaluation of the goal and meaning of life, and their own sense of achievement and satisfaction. It includes two parts, i.e., the presence of meaning and the search for meaning [35, 36]. Therefore, as this concept relates to the attitude and competencies toward death, the study introduced the meaning of life in order to investigate the mechanisms that enhance nurses’ CDC further.

The meaning of life has also been emphasized by life and death educators in recent years. Due to the different measuring instruments, the scores of nurses’ meaning of life also differed; but in general, it was at the moderate level [37, 38]. A significant correlation was also found between nurses’ sense of meaning in life and their attitude toward death. The more the positive attitude toward death, the stronger the sense of meaning in life was [37]. A study conducted in Hong Kong revealed that the higher the ability of professionals to engage in death work, the higher their quality of life, including the acceptance of death and sense of meaning in life [39]. The sense of meaning in life was also found to be positively correlated with CDC, and the impact of the presence of meaning in life on CDC was even greater than that of the experience of relative’s death [40]. A study has shown that death education can improve college students’ sense of life meaning [34].

### Aims

**Fig. 1:**
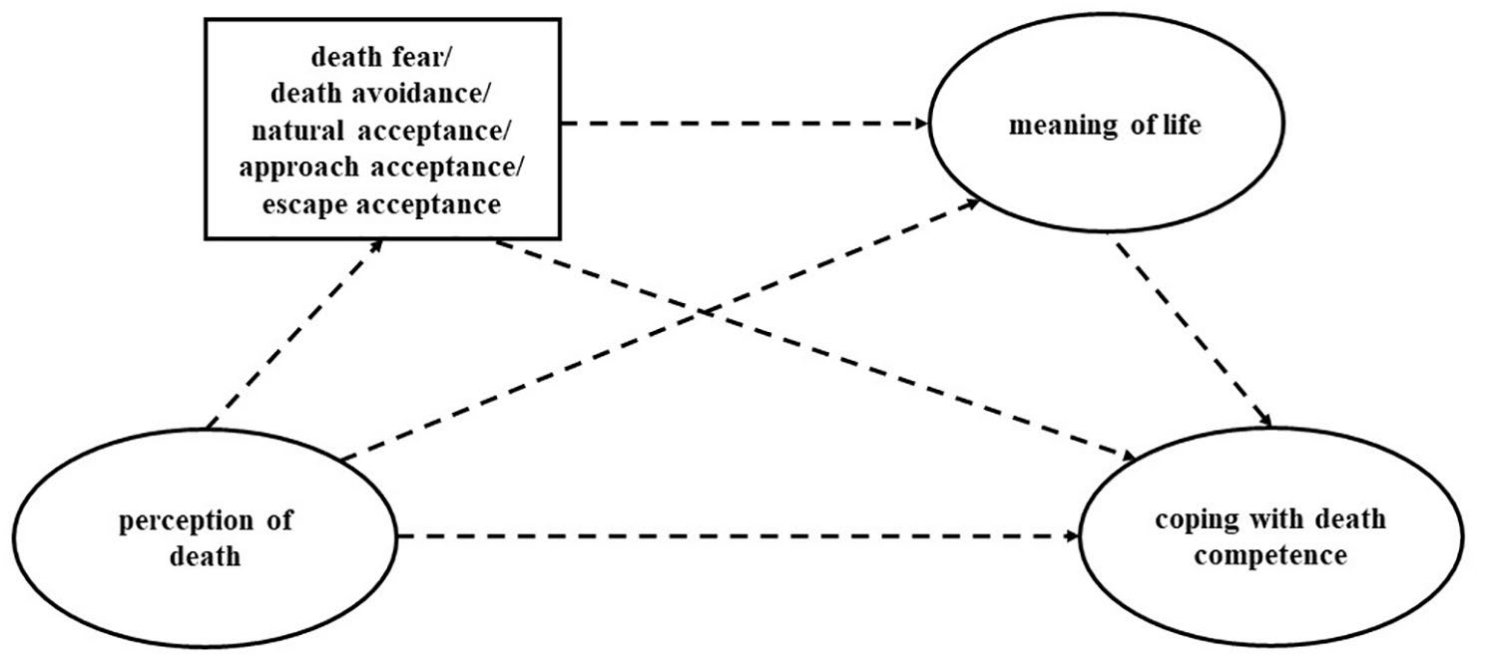
The conceptual model

Based on the above theoretical analysis and the results of previous studies, we considered that attitude toward death and meaning of life play a chain mediating role in nurses’ death perception and CDC. Thus, we proposed the following hypotheses (Fig. 1):

#### Hypothesis 1

Nurses’ perception of death would positively predict their CDC.

#### Hypothesis 2

Nurses’ attitude toward death would have a mediating role in the perception of death and CDC.

#### Hypothesis 3

Nurses’ sense of meaning in life would have a mediating role in the perception of death and CDC.

#### Hypothesis 4

Nurses’ attitude toward death and sense of meaning in life would have a chain mediating role in the perception of death and CDC.

## The study

### Design

This is a cross-sectional design study.

### Participants

Participants were recruited by convenience sampling across six tertiary hospitals in different cities in Hunan Province. According to the empirical rule, the sample number is generally 10 ~ 20 times the number of items; and therefore the final sample size was 539 ~ 1176, taking into account a 10% ~ 20% missed visit rate. In addition, since there are nearly 280,000 registered nurses in Hunan Province, we distributed as many questionnaires as possible within the allowed range to ensure the representativeness of the data. The research setting of this study was inpatient wards. The inclusion criteria were as follows: (1) agree to participate in the study and give informed consent; (2) on-the-job nurses in front-line clinical positions. To ensure the objectivity and authenticity of the research results, the scale was completed anonymously on the Internet. Initially, 841 questionnaires were collected; and finally, 786 valid questionnaires (the effective rate was 93.5%) were left.

### Data collection and ethical considerations

This study was conducted in Hunan Province, China, from October to November 2021. Before data collection, we contacted the nursing managers from six tertiary hospitals and explained to them the details of the study, asking for their assistance with the survey. In this study, online questionnaire platform (https://www.wjx.cn/) was used for investigation. The link of online questionnaire was distributed to the WeChat group of nurses working in the above-mentioned six hospitals. The questionnaire was preceded by an explanation of the study purpose. It firstly informed the participants of the time needed to complete the questionnaire (about 10–15 min), the voluntariness of their participation in the study, the possibility to withdraw at any time, the anonymity and confidentiality of their information with the use only for this study, as well as the informed consent being tacitly granted once the questionnaire was completed. After data collection, the data were strictly screened by two researchers, with the exclusion of response duration < 200s, the number of the same options > 80%, and self-contradictory answers. This study has been approved by Ethical Review Committee of Nursing and Behavioral Medicine Research, School of Nursing, Central South University (approval NO. E2021116). The study follows STROBE Statement.

### Instruments

In addition to participants’ personal information, such as age, gender, marital status, years of experience, and job title, the online questionnaire used in this study consisted of four parts.

The first part was a self-made questionnaire on the perception of death, including the two following aspects: understanding of death, and understanding of good death. The number of items in each part was 16 and 10 respectively; and the Cronbach’s α was 0.777, and 0.864 respectively. The first part examines the understanding of death from four dimensions: physical, spiritual, social, and cultural. The second part investigates the understanding of good death from many perspectives like longevity, illness, accidents, suffering, and regret. The Likert5 scoring system was adopted, where a score of 1–5 corresponded from “totally disagree” to " totally agree”. The final score is the sum of the scores of each item divided by the total number of items. Higher scores indicate higher levels of death perception.

The second part was the Coping with Death Scale (CDS), which was developed by Bugen [41] and compiled in Chinese by a Taiwan scholar [42]. This scale is widely used to measure the CDC. The Cronbach’s α of the scale is 0.913. A total of 30 items were scored by Likert7 scale, where a score of 1–7 corresponded from “totally disagree” to “totally agree”. The higher score reflects the higher CDC.

The third part was The Meaning in Life Questionnaire (MLQ), which was compiled [36] to evaluate the existence of life and find the meaning of life. The Chinese version of MLQ adopted in the study [43] is divided into two subscales, i.e., The Presence of Meaning (MLQ- P) and The Search for Meaning (MLQ-S), with each subscale containing 5 items. The score of 1–7 corresponded from “totally disagree” to “totally agree”. The α coefficients for internal consistency of the total scale, MLQ-P, and MLQ-S, were 0.830, 0.842, and 0.828, respectively; and the test-retest reliability was 0.639, 0.746, and 0.558, respectively.

The fourth part was the Chinese version of the Death Attitude Profile-Revised (DAP-R) [44]. The Cronbach’s α coefficient of the total scale for nurses was 0.875, showing relatively high internal consistency, homogeneity and reliability. The scale includes 32 items and 5 dimensions, i.e., fear of death, death avoidance, natural acceptance, approach acceptance, and escape acceptance. Likert 5-point scoring method was adopted, with a total score ranging from 32 to 160 points. The score of this part is the sum of the scores of each item divided by the number of items. The higher the score, the more positive the attitude of the participants is toward the death.

### Data analysis

SPSS26.0 was used to make statistical analysis; mean ± standard deviation was to describe the level of each variable; and Person, to explore the relationship among variables. AMOS26.0 was applied to establish the structural equation model among the factors related to CDC, so as to explore the internal relationship between the factors and their working paths. The differences were considered statistically significant at p < 0.05. The conceptual model is shown in Fig. 1. Factor algorithm [45] was introduced for packaging CDC, and internal consistency method [46] for packaging perception of death and meaning of life. We evaluate the fitness of the model by chi-square values (χ^2^/df), the comparative fit index (CFI), the Tucker-Lewis fit index (TLI), the root mean square error of approximation (RMSEA), and the standardized root mean square residual (SRMR). We use bootstrap Maximum Likelihood (ML) for 5,000 times within the 95% confidence interval to test the significance of the direct, indirect, and total effects of the model; and the initial model was further modified according to the model Modification Index (MI).

## Results

### Common method biases tests

Herman’s single factor test for common method bias was used [47]. The results showed that the maximum factor variance explanation degree was 19.75% (< 40%), proving no obvious common method bias in this study.

### Participants’ characteristics

As shown in Table 1, a total of 786 nurses effectively participated in the study, including 8 males (1.0%) and 778 females (99.0%). The participants aged 32.83 ± 7.51 years and their working years was 11.69 ± 8.28. Besides, 26.0% of the participants were unmarried, 72.0% married, 1.9% divorced and 0.1% windowed. Finally, 14.1% of the participants were nurse, 29.9% senior nurse, 45.3% nurse in charge, 9.8% deputy chief nurse and 0.9% chief nurse.

**Table 1:** Demographic characteristics of nurses(*N* = 786)

Variable		N	%
Gender	Male	8	1.0
	Female	778	99.0
Marital status	Unmarried	204	26.0
	Married	566	72.0
	Divorced	15	1.9
	Windowed	1	0.1
Job title	Nurse	111	14.1
	Senior nurse	235	29.9
	Nurse in charge	356	45.3
	Deputy chief nurse	77	9.8
	Chief nurse	7	0.9

### Descriptive statistics and correlations of perception of death, CDC, meaning of life, and attitude toward death

Table 2 shows the average, standard deviation, and correlation coefficient of each variable in this study. By comparing the scores of DAP-R five dimensions, nurses’ attitude toward death was found to be more inclined to natural acceptance. A positive correlation was found among death cognition, CDC, sense of meaning in life, and attitude toward death. The five dimensions of attitude toward death were all positively correlated with the perception of death. Natural acceptance, approach acceptance, and escape acceptance were positively correlated with CDC; while death escape, natural acceptance, approach acceptance were positively correlated with the meaning of life.

**Table 2:** Descriptive statistics and variable correlations(*N* = 786)

	1(r)	2(r)	3(r)	4(r)	M ± SD(score)
1 Perception of death	1.00	—	—	—	3.45 ± 0.47
2 CDC	0.229^**^	1.00	—	—	125.39 ± 23.88
3 Meaning of life	0.168^**^	0.399^**^	1.00	—	48.33 ± 8.66
4 Attitude toward death	0.332^**^	0.247^**^	0.092^**^	1.00	95.96 ± 16.06
5 Death fear	0.196^**^	-0.031	-0.026	—	2.80 ± 0.70
6 Death avoidance	0.161^**^	-0.006	0.128^**^	—	3.06 ± 0.73
7 Natural acceptance	0.245^**^	0.362^**^	0.238^**^	—	3.81 ± 0.59
8 Approach acceptance	0.332^**^	0.207^**^	0.078^*^	—	2.79 ± 0.71
9 Escape acceptance	0.175^**^	0.215^**^	-0.028	—	2.82 ± 0.83

** *p* < 0.01

* *p* < 0.05

### Mediating effect analysis

**Fig. 2:**
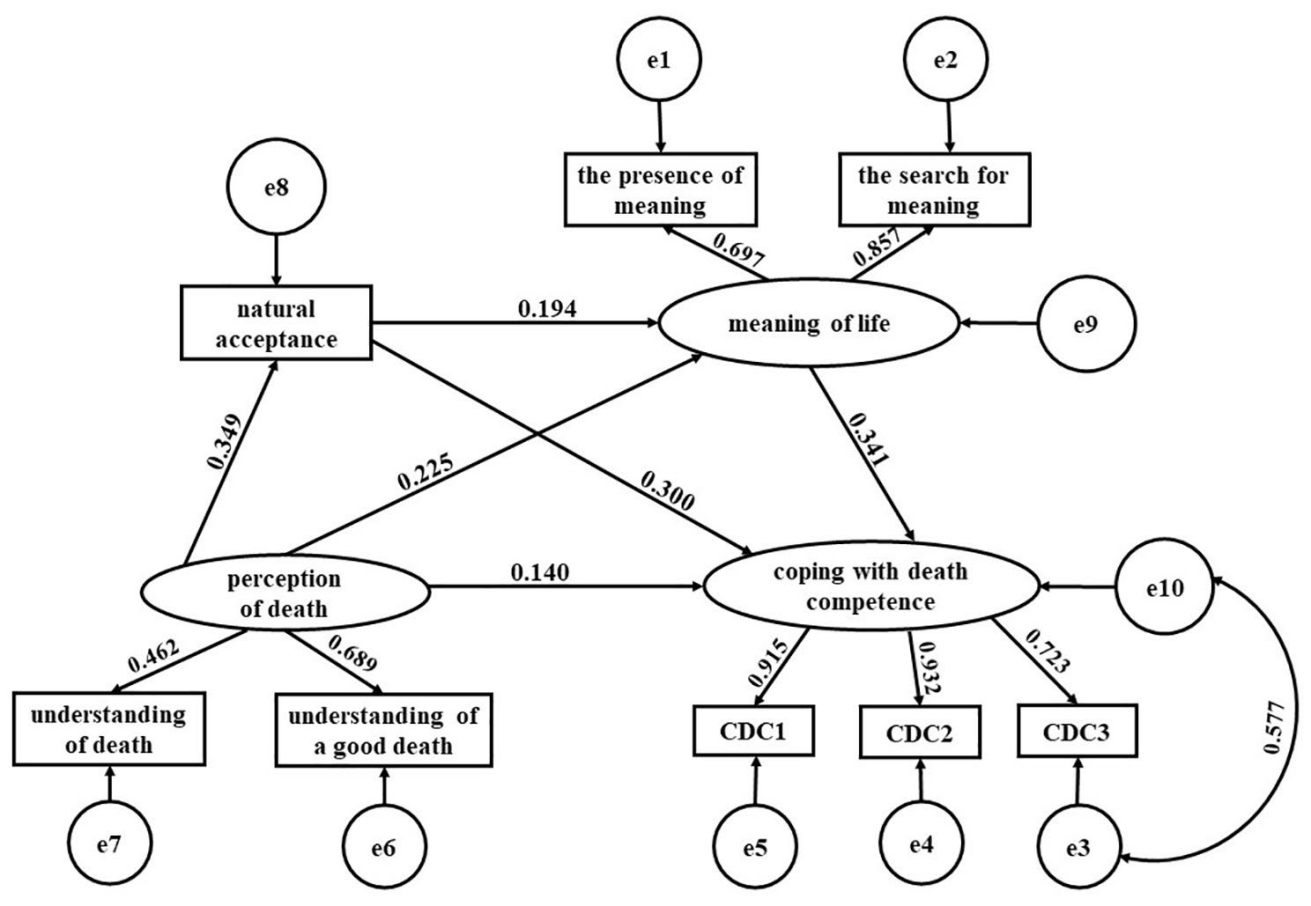
Structural equation model figure

Based on the KAP and a large number of literature review, combined with the results of correlation analysis, the structural equation model was initially constructed to test the mediating effect of natural acceptance, approach acceptance and the meaning of life between the perception of death and CDC. As the model results showed that the mediating effect of approach acceptance and the mediating chain effect of approach acceptance and meaning of life were not significant, only the results of natural acceptance are reported. The initial results of the model fit test were: χ^2^/df = 7.448, SRMR = 0.029, GFI = 0.967, TLI = 0.942, RMSEA = 0.091, and therefore further revision was needed. Adjustments were made according to the MI values and the logical correlations of each variable. The adjusted final model is shown in Fig. 2. Its parameters are as follows: χ^2^/df = 3.991, SRMR = 0.028, GFI = 0.982, TLI = 0.973, RMSEA = 0.062 (The ideal values are < 3, < 0.05, > 0.9, > 0.9 and < 0.08, respectively), and therefore the model fits well. The model showed that the positive predictive ability exists in perception of death to natural acceptance (*β* = 0.349, *p* < 0.001) and sense of meaning in life (*β* = 0.225, *p* < 0.01); in natural acceptance (*β* = 0.300, *p* < 0.001) and sense of meaning in life (*β* = 0.341, *p* < 0.01) to CDC; in natural acceptance to sense of meaning in life (*β* = 0.194, *p* < 0.01). And perception of death had direct predictive ability to CDC (*β* = 0.140, *p* < 0.05). The model is shown in Fig. 2.

The Bootstrap method was used to test the confidence interval estimation. 5,000 samples were repeated, and 95% confidence intervals were calculated. The results showed that the 95% confidence intervals of the three paths did not include 0, which was statistically significant, indicating that perception of death mainly affected CDC through the natural acceptance and the sense of meaning in life. The test results are shown in detail in Table 3.

**Table 3:** Mediation effect test results

Path	Effect	SE	95%CI	Relative mediation effect
**Direct effect**				
Perception of death→CDC	0.140	0.067	0.021 ~ 0.287	40.70%
**Indirect effect**				
Perception of death→Natural acceptance→CDC	0.105	0.023	0.061 ~ 0.152	30.52%
Perception of death→Meaning of life→CDC	0.077	0.029	0.024 ~ 0.137	22.38%
Perception of death→Natural acceptance→Meaning of life	0.023	0.009	0.008 ~ 0.042	6.68%
**Total effect**	0.344	0.066	0.214 ~ 0.471	

## Discussion

This study identified the mediating pathways by which perception of death influences CDC, and verified all the hypotheses of the study. The findings showed that natural acceptance and meaning of life play mediating roles in the relationship between perception of death and CDC.

It was found that death perception was significantly and positively correlated with CDC, indicating that higher levels of death perception were associated with higher levels of CDC, which is the same as the previous results [48]. A study has shown that death education can improve CDC, within which the most basic and pervasive education component is perception of death [49]. A study on Chinese emergency department nurses also showed that death education enhanced perceptions of effective behavioral responses in dealing with sudden death, improving the quality of their work [50]. According to KAP, acquiring death-related knowledge is the foundation of CDC change, and death education is currently the most common way for acquisition. Death education in China started relatively late and developed slowly due to the traditional Chinese naive pragmatism and the long-standing cultural avoidance of “death”. This has to some extent affected the development of Chinese people’s CDC and hospice care, and is not conducive to the improvement of the quality of death in China.

This study found that natural acceptance played a significant mediating role in the relationship between perception of death and CDC, indicating that higher levels of death perception enhance nurses’ CDC by improving natural acceptance of attitude toward death. This further verifies the KAP: taking the example of nurses, only when they acquire death-related knowledge with a positive attitude and create a strong sense of responsibility, can they gradually form relevant beliefs. Only when their death-related knowledge is upgraded to belief, it is possible to adopt a positive attitude toward death, thus improving their behavior in dealing with death-related events. Natural acceptance holds that death is an inevitable fact of life; it neither fears nor welcomes the arrival of death. It is a positive attitude toward death, which the scholars hope to develop in the educated through death education [51]. A study has reported a significant relationship between the view of death among Chinese nurses and their attitude toward nursing dying patients [23]. Their perception of death may affect their attitude toward their own death and death of others [52]. Different levels of knowledge and skills may result in different attitudes toward death, as well as different strategies and behaviors in dealing with death-related events [53]. A positive attitude toward death has a positive impact on the care of the dying [54]. Cardoso et al. [53] found that during the period after the COVID-19 pandemic, specialist nurses tended to accept death more naturally than other nurses, with their knowledge and skills as important factors in promoting a positive attitude toward death, resulting in positive behaviors and strategies to deal with disease and death. Our results fit well with the above studies, that is, perception of death indirectly influences CDC through natural acceptance attitudes.

In addition, the mediating effect of meaning of life between perception of death and CDC was also significant. Death is a part of life; life and death are related to and influenced by each other. Due to the constraints of traditional Chinese culture, death education in China is often carried out only as a part of life education. In a life education course for medical students at a medical college in Jiangsu Province, death-related course took up only 90 min (out of a total of 960 min) [55]. It is necessary to develop meaning in life by the awareness of the fact that human beings are destined to die, which in turn will facilitate the development of the CDC [56]. The results of the study showed that the meaning of life was significantly and positively correlated with perception of death, CDC, and natural acceptance, which is the same as the previous studies. Gao et al. [37] found a significant positive correlation between nurses’ sense of meaning in life and natural acceptance through surveying 464 nurses in tertiary hospitals. Miller et al. [40] conducted a survey of 277 participants taking a MOOC on the topic of death in Australia and found the closest relationship between the presence of meaning in life and a higher level of CDC. In the process of thinking about death, nurses with a higher level of death perception will indispensably expand “death” to “life” and reflect on life goals and the meaning of life. While nurses with a strong sense of life meaning will be more aware of the value of their existence and life goals in this process, with stronger psychological motivation to cope with death in clinical work and reduce the impact of negative emotions on themselves, which are conducive to the improvement of CDC.

Finally, there was a chain mediating effect of natural acceptance and meaning of life between perception of death and CDC. Improving the perception death and CDC enhances nurses’ death awareness and their quality of end-of-life care. It also helps popularize death education for the general public, the hospice care, and healthy aging. The natural acceptance of death as an objective existence and the finite nature of life motivates people to more fully enjoy the beauty of life and explore its essence. It stimulates their sense of accomplishment with their own existence and goal achievement, which in turn mobilizes their internal and external strength to cope with the negative emotions brought about by death-related events and to improve communication with bereaved families. Against the current backdrop of COVID-19 pandemic, the large numbers of cases and deaths have posed a major challenge to nurses with compassion fatigue, moral distress, burnout, and posttraumatic stress syndrome, which is not conducive to their physical and mental health as well as care for patients and their families [57, 58]. There is an urgent need for nurses to strengthen their learning about death, establish positive attitude toward death, as well as enhance their sense of the meaning of life and CDC.

Our results provide a basis for effectively improving nurses’ CDC. Nursing educators and administrators can set up advanced course in death education and clearer educational purposes to improve nurses’ CDC more scientifically based on these results. In addition, the effectiveness of death education can also be tested by natural acceptance attitude towards death and meaning of life. And the course content can be adjusted accordingly to achieve the ultimate goal of improving CDC. Future studies can focus on more mechanisms and paths affecting CDC, providing theoretical basis and empirical evidence for formulating more scientific education strategies.

## Limitations

Although the present study used rigorous methods, the sample size was large, and the structural equation model was used to explore the mediation effect, there are still some limitations. Firstly, the convenience sampling method was adopted in this study to select some samples from Hunan Province, China, which may not represent all nurses in mainland China, thus limiting the extensibility of the results. Multi-center investigation and research should be carried out in the future to overcome this limitation. Secondly, this study was included self-reported questionnaire with a cross-sectional study which may be some limitations such as reporting bias.

## Conclusion

The results showed that the relationship between nurses’ perception of death and CDC was mainly mediated by natural acceptance attitude and sense of meaning in life. Therefore, according to the results, multi-level death education courses could be designed to progressively improve the CDC of nurses from the aspects of knowledge, emotion, attitude, belief, and ability.

## Data Availability

The datasets used and/or analyzed during the current study are available from the corresponding author on reasonable request.
